# Angiotensin II Induces Differentiation of Human Neuroblastoma Cells by Increasing MAP2 and ROS Levels

**DOI:** 10.1155/2021/6191417

**Published:** 2021-06-14

**Authors:** Bryan Jael Collazo, Dariana Morales-Vázquez, Jaylene Álvarez-Del Valle, Javier E. Sierra-Pagan, Juan Carlos Medina, Jarold Méndez-Álvarez, Yamil Gerena

**Affiliations:** Department of Pharmacology and Toxicology, School of Medicine, University of Puerto Rico, Medical Sciences Campus, PO Box 365067, San Juan, Puerto Rico 00936-5067

## Abstract

**Introduction:**

The roles of angiotensin II (Ang II) in the brain are still under investigation. In this study, we investigated if Ang II influences differentiation of human neuroblastoma cells with simultaneous activation of NADPH oxidase and reactive oxygen species (ROS). Moreover, we investigated the Ang II receptor type involved during differentiation.

**Methods:**

Human neuroblastoma cells (SH-SY5Y; 5 × 10^5^ cells) were exposed to Ang II (600 nM) for 24 h. Differentiation was monitored by measuring MAP2 and NF-H levels. Cell size and ROS were analyzed by flow cytometry, and NADPH oxidase activation was assayed using apocynin (500 *μ*M). Ang II receptors (ATR) activation was assayed using ATR blockers or Ang II metabolism inhibitors (10^−7^ M).

**Results:**

(1) Cell size decreased significantly in Ang II-treated cells; (2) MAP2 and ROS increased significantly in Ang II-treated cells with no changes in viability; (3) MAP2 and ROS decreased significantly in cells incubated with Ang II plus apocynin. (4) A significant decrease in MAP2 was observed in cells exposed to Ang II plus PD123.319 (AT2R blocker).

**Conclusion:**

Our findings suggest that Ang II influences differentiation of SH-SY5Y by increasing MAP2 through the AT2R. The increase in MAP2 and ROS were also mediated through NADPH oxidase with no cell death.

## 1. Introduction

The renin angiotensin system (RAS) in the brain has been highlighted as having a role in the pathophysiology of several neurodegenerative diseases. It is known that Ang II, its major effector peptide, plays an important role in oxidative stress, CNS inflammation, neuronal injury, cellular senescence, and cell differentiation [1–4]. Emerging evidence supports that Ang II is an important mediator of cellular differentiation in different mammalian cells [5–9]. Wu et al. and Zheng et al. showed that Ang II promotes mouse embryonic stem cell differentiation into cardiomyocytes as determined by increased expression levels of cardiac markers such as GATA4 and troponin-T [5], or into smooth muscle cells as determined by increased expression levels of smooth muscle markers such as calponin [6]. In addition, Kim et al. revealed that Ang II acts as a differentiator factor of mice hematopoietic stem cells into myeloid cells analyzed by increased expression levels of chemokine receptor type 2 (CCR2) [7]. Ang II can also induce neuronal differentiation with neurite outgrowth on PC12W cells, a clone derived from a pheochromocytoma tumor of the rat adrenal medulla (PC12) used in several studies to monitor neuronal differentiation [8, 9]. Cell differentiation was confirmed by increased levels of MAP2 and *β*-tubulin proteins induced by Ang II.

It is also well known that Ang II induces ROS production in different mammalian cells [10–12]. El Bekay et al. found that Ang II enhances ROS production in human neutrophils when incubated with concentrations ranging from 10 to 1000 nM for a period of 1 to 10 min [10]. Other reports have documented that Ang II increases superoxide levels in rat mesangial cells exposed to Ang II (10^−8^-10^−5^ M) and in human astrocytes at 100 nM [11, 12]. Although these studies support that Ang II has a role in oxidative stress activation, the relationship between ROS production and differentiation induced by the peptide in human neuroblastoma cells remains to be elucidated. It is known that ROS themselves are able to promote differentiation in many mammalian cells. Tsatmali et al. revealed that embryonic rat cortical cells expressing higher levels of ROS induced by fibroblast growth factor 2 (FGF2) differentiate into two types, large pyramidal-like neurons and smaller neurons expressing nuclear calretinin; however, neurons with low levels of ROS after FGF2 removal differentiate into neurons, oligodendrocytes, and astrocytes in clonal cultures [13, 14]. In addition, Katoh et al. showed that hypoxia enhances the signal for neuronal differentiation by producing ROS in PC12 cells [15].

Another important aspect to consider in ROS activation induced by Ang II is the possible mechanism employed by the peptide that enhances the production of these oxidative stress mediators. Several studies revealed that Ang II stimulates the production of ROS through the activation of NADPH oxidase in skeletal muscle [16], cardiovascular system [17], and brain [18, 19]. Kazama et al. reported that Ang II increases ROS production in isolated mice cerebral microvessels via a gp91phox containing NADPH oxidase [18]. Moreover, Sun et al. found that Ang II increases ROS levels through NADPH oxidase activation in rat neuronal cells from the hypothalamus and brain stem areas [19]. Although these reports support that NADPH oxidase is involved in Ang II-induced ROS production in different cells, no clear data exist about their possible role in differentiation of human neuroblastoma cells.

Based on these observations, we decided first to investigate if Ang II influences the morphology and differentiation of human neuroblastoma cells and the production of intracellular ROS without affecting the cell viability. In addition, we analyzed if the generation of neuroblastoma MAP2 expression and ROS levels were mediated through the activation of NADPH oxidase. Furthermore, we investigated the type of angiotensin II receptor (ATR) involved in the Ang II-induced neuroblastoma differentiation.

## 2. Methods

### 2.1. Cell Size Analysis by Microscopy and Flow Cytometry

Human neuroblastoma cells (SH-SY5Y) were cultured (5 × 10^5^ cells) in Minimum Essential Medium (MEM) supplemented with 10% fetal bovine serum at 37°C with 5% CO_2_. When the cells reached a 70% confluence, they were exposed to Ang II (600 nM), and cell size was visualized using the EVOS® FLoid Cell Imaging Station (Life Technologies, San Juan, PR) equipped with a 20x fixed objective and a monochrome high-sensitivity interline CCD camera. The images were captured in bright-field mode using the FLoid software version 1.4 (Life Technologies, San Juan, PR). Cell size was also analyzed by flow cytometry.

### 2.2. Analysis of MAP2 and NF-H Levels in Cells Exposed to Ang II

Neuroblastoma cells were incubated with Ang II (600 nM) for 24 h and MAP2 and NF-H were labeled with monoclonal fluorescent antibodies specific for those cytoskeletal protein markers in dendrites and axons, respectively. Briefly, cells were permeabilized using BD Cytofix/Cytoperm™ Fixation/Permeabilization Solution Kit (BD Biosciences, San Jose, CA) and then incubated with Alexa Fluor 488 anti-MAP2 (1 : 1000) or Alexa Fluor 647 anti-NF-H (1 : 1000) for 1 h. Both antibodies were purchased from BioLegend (San Diego, CA). Samples were analyzed by flow cytometry.

### 2.3. Effects of Ang II in ROS Production and Apoptosis Assay

Neuroblastoma cells were exposed to Ang II (600 nM) for 24 h. ROS were stained using the Oxidative Stress Detection Reagent (Green Fluorescent) as described by the manufacturer (ENZO Life Sciences, Farmingdale, NY). For the apoptosis assay, SH-SY5Y were resuspended in annexin-binding buffer (10 mM HEPES, 140 mM NaCl, and 2.5 mM CaCl_2_, pH 7.4) at a concentration of 1 × 10^6^ cells/mL. Then, 5 *μ*L of Annexin-V fluorescein isothiocyanate (FITC) conjugate and 5 *μ*L of propidium iodide (PI) (4 mg/mL) were added to 100 *μ*L of cell suspension and incubated for 15 min at room temperature. Finally, 400 *μ*L of annexin-binding buffer were added and samples were analyzed by flow cytometry.

### 2.4. MAP2 Expression Levels and ROS Production Induced by Ang II after NADPH Oxidase Inhibition

Neuroblastoma cells were incubated with Ang II (600 nM) or Ang II plus apocynin (500 *μ*M; NADPH oxidase inhibitor) for 24 h in order to investigate if the neuroblastoma differentiation and generation of ROS induced by the peptide were mediated through NADPH oxidase. Untreated cells and those exposed to the drug alone were used as controls. MAP2 levels or intracellular ROS levels were labeled with a fluorescent monoclonal antibody or reagent, respectively, as described above and then analyzed by flow cytometry.

### 2.5. MAP2 Expression Levels after Ang II Receptors Blockers or Ang II Metabolism Inhibitors

To investigate if the effects of Ang II or its metabolites [Ang IV or Ang-(1–7)] on MAP2 were mediated through its binding to the AT1, AT2, AT4, or Ang-(1–7) receptors, cells were incubated with Ang II (600 nM) in the presence or absence of Losartan (selective AT1R blocker; 10^−7^ M), PD123.319 (selective AT2R blocker; 10^−7^ M), AP-N/CD13, 4-tert-butylphenyl hydrogen 1-aminopropylphosphonate (Aminopeptidase N inhibitor; 10^−7^ M), or MLN-4760 (ACE2 Inhibitor; 10^−7^ M) for 24 h. Untreated cells and those exposed to the drugs alone were used as controls. All drugs were purchased from Millipore Sigma (Saint Louis, MO). MAP2 levels were analyzed using a specific fluorescent monoclonal antibody as described above and then analyzed by flow cytometry.

### 2.6. Flow Cytometry

All flow cytometric analyses were carried out using a FACSAria cytometer (BD Biosciences, CA). The FACSDiva software (BD Biosciences, CA) was used for data acquisition and multivariate analysis. SH-SY5Y cells were gated in forward/side scatter (FSC/SSC) dot plots, and cell size was determined by obtaining the median peak channel from the FSC histogram. Intracellular ROS, MAP2 levels, or Annexin V were measured in the FL1 (band pass filter 525 nm), PI was measured in the FL3 (long pass 670 nm), and NF-H levels were analyzed in the FL4 (bandpass filter 661 nm) channels. Data on scatter parameters and histograms were acquired in log mode. Ten thousand events were evaluated for each sample, and the median peak channel obtained from the FL1 and FL4 histograms was used to determine the intracellular ROS, MAP2, and NF-H levels from neuroblastoma cells. The percent frequency of healthy, early apoptotic, late apoptotic, or necrotic cells in dot plots was used to determine the cell viability.

### 2.7. Statistical Analysis

Data were expressed as mean ± SEM. Student's *t*-test and ANOVA were used. Normality of populations and homogeneity of variances were tested before each ANOVA. Differences were considered significant when *p* < 0.05. Data were normalized to the mean of the untreated group.

## 3. Results

### 3.1. Visualization of Morphology after Ang II Treatment

**Figure 1:**
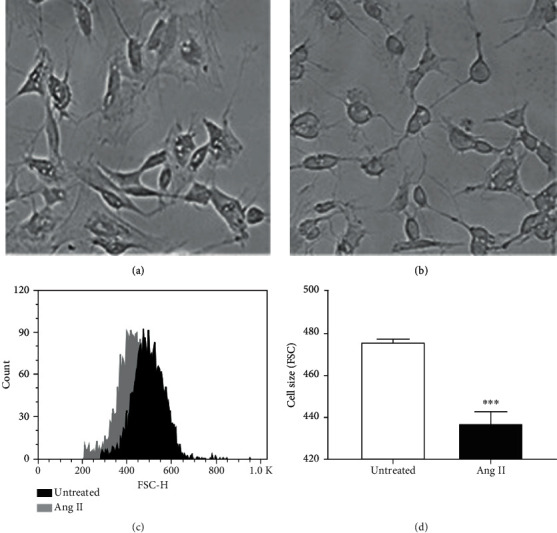
Morphological changes in neuroblastoma cells after Ang II treatment. Cells were incubated with Ang II (600 nM) for 24 h, and cell size changes were visualized by bright-field microscopy. Changes in cell size were also analyzed by flow cytometry. (a) Untreated neuroblastoma cells visualized in bright-field. (b) Ang II-treated neuroblastoma cells visualized in bright field. Changes in cell size and shape (b) were observed in cells exposed to Ang II when compared to untreated (a). (c) Representative flow cytometric histogram of untreated (dark peak) and Ang II-treated (gray peak) neuroblastoma cells. (d) Cell size significantly decreased after incubation with Ang II (dark bar) when compared to untreated (open bar). Each bar represents the average of five experiments. Vertical lines at each bar represent SEM (^∗∗∗^*p* < 0.001). FSC: forward scatter.

Human neuroblastoma cells exposed to Ang II (600 nM) for 24 h were visualized in bright-field microscopy. Morphological changes in cell size and shape were observed under the microscope after cells were incubated with Ang II (Figure 1(b)) when compared to untreated (Figure 1(a)). The cell soma size was reduced by Ang II treatment with elongated neurites. Histograms obtained by flow cytometry showed the differences in size of neuroblastoma cells prior to and after incubation with Ang II (Figure 1(c)). A significant decrease in size (*p* < 0.001) was observed after exposure to Ang II for a period of 24 h (Figure 1(d)).

### 3.2. MAP2 and NF-H Levels in Ang II-Treated Neuroblastoma Cells

**Figure 2:**
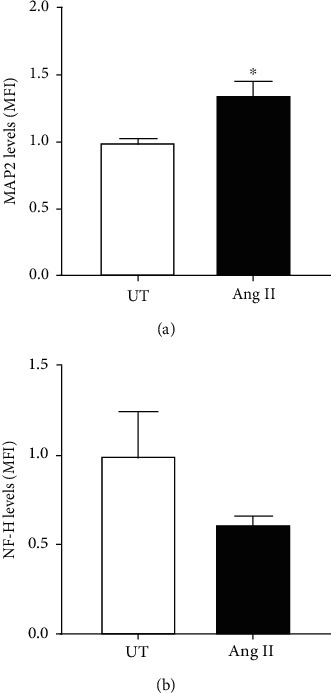
Effect of Ang II-treated neuroblastoma cells on MAP2 and NF-H expression levels. Cells were incubated with Ang II (600 nM) for 24 h, and the levels of MAP2 and NF-H proteins were measured by flow cytometry using specific monoclonal antibodies. (a) MAP2 levels increased significantly when the cells were incubated with Ang II compared to untreated. (b) No significant differences were observed in the levels of NF-H when the cells were incubated with Ang II compared to untreated. Each bar represents the average of four experiments. Vertical lines at each bar represent SEM (^∗^*p* < 0.05). MFI: mean fluorescence intensity.

Cells were incubated with Ang II (600 nM) for 24 h, and MAP2 and NF-H levels were measured by flow cytometry to monitor neuroblastoma differentiation. Our data revealed that MAP2 levels increased significantly when the cells were exposed to Ang II (1.36 ± 0.11 MFI; *p* < 0.05) compared to untreated (1.00 ± 0.05 MFI) (Figure 2(a)). No significant differences were observed in the levels of NF-H (0.62 ± 0.06 MFI) when the cells were exposed to Ang II compared to untreated (1.00 ± 0.26 MFI) (Figure 2(b)).

### 3.3. Ang II-Induced ROS Levels and Cell Viability

**Figure 3:**
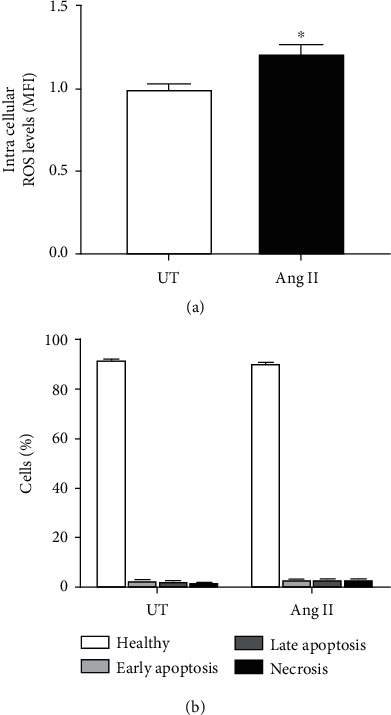
Effect of Ang II in the generation of intracellular ROS and cell viability. Human neuroblastoma cells were cultured in the presence of Ang II (600 nM) for 24 h, and intracellular ROS levels and the percentage of healthy, apoptotic, or necrotic cells were analyzed by flow cytometry. (a) Ang II increased significantly the intracellular ROS levels in neuroblastoma cells when compared to untreated. (b) No significant differences were observed in the percentage of healthy, apoptotic, or necrotic cells in Ang II-treated neuroblastoma cells when compared to untreated. Each bar represents the average of three experiments. Vertical lines at each bar represent SEM (^∗^*p* < 0.05). MFI: mean fluorescence intensity.

Neuroblastoma cells were incubated with Ang II at 600 nM for 24 h, and the levels of intracellular ROS and the percentage of healthy, apoptotic, or necrotic cells were quantified by flow cytometry. Our data revealed a significant increase in ROS levels when cells were exposed to Ang II at 600 nM (1.22 ± 0.06 MFI; *p* < 0.05) compared to untreated (1.00 ± 0.04 MFI) (Figure 3(a)). On the other hand, our results showed that the percentage of healthy, apoptotic, or necrotic cells were similar in Ang II-treated cells and nontreated groups (Figure 3(b)).

### 3.4. Effect of NADPH Oxidase Inhibition in Ang II-Induced MAP2 Expression Levels and ROS Production

**Figure 4:**
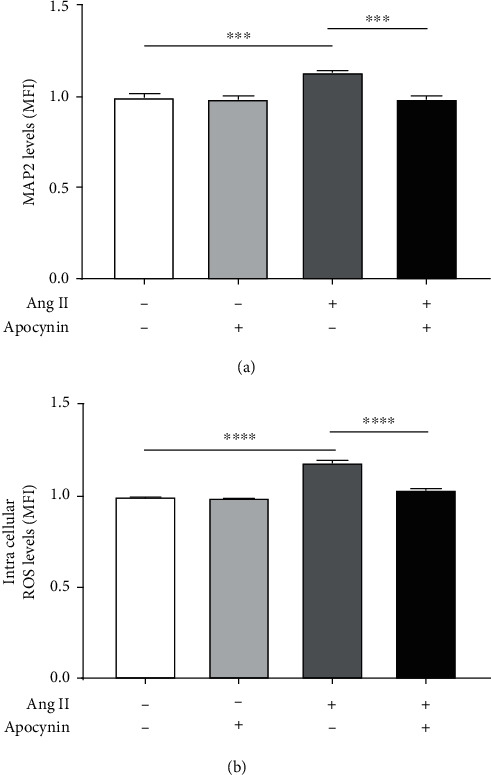
Effect of apocynin on neuroblastoma MAP2 expression levels and ROS production induced by Ang II. Cells were incubated with Ang II (600 nM) alone or Ang II plus apocynin (500 *μ*M) for 24 h, and the levels of MAP2 and intracellular ROS were quantified by flow cytometry. A significant increase was observed on MAP2 (a) and intracellular ROS levels (b) when the cells were incubated with Ang II alone (dark gray bar) compared to untreated (open bar). Both MAP2 and intracellular ROS levels decreased significantly when the cells were exposed to Ang II plus apocynin (dark bar) compared to those exposed to Ang II alone (dark gray bar). No significant differences were observed in the levels of MAP2 (a) or intracellular ROS (b) between cells that were exposed to Ang II plus apocynin (dark bar) and untreated (open bar) or apocynin alone (light gray bar). Each bar represents the average of four experiments. Vertical lines at each bar represent SEM (^∗∗∗^*p* < 0.001; ^∗∗∗∗^*p* < 0.0001). MFI: mean fluorescence intensity.

Cells were incubated with Ang II (600 nM) in the presence or absence of apocynin (500 *μ*M) for 24 h, and MAP2 or intracellular ROS levels were measured by flow cytometry. Our data revealed that MAP2 levels increased significantly when the neuroblastoma cells were exposed to Ang II alone (1.14 ± 0.01 MFI) compared to untreated (1.00 ± 0.02 MFI). On the other hand, a significant decrease was observed in MAP2 levels when the cells were incubated with Ang II plus apocynin (0.99 ± 0.02 MFI; *p* < 0.001) compared to cells incubated with Ang II alone. No significant changes were observed in MAP2 levels between neuroblastoma cells incubated with Ang II plus apocynin (0.99 ± 0.02 MFI) and apocynin alone (0.99 ± 0.02 MFI) (Figure 4(a)). Similarly, ROS levels increased significantly when the cells were incubated with Ang II alone (1.19 ± 0.02 MFI) compared to untreated (1.00 ± 0.01 MFI), and a significant decrease was observed when the cells were exposed to Ang II plus apocynin (1.04 ± 0.01 MFI; *p* < 0.0001) compared to cells exposed to Ang II alone. In addition, no significant changes were observed in ROS levels between cells exposed to Ang II plus apocynin (1.04 ± 0.01 MFI) and apocynin alone (0.99 ± 0.01 MFI) (Figure 4(b)).

### 3.5. Effect of Ang II Receptors Antagonism on MAP2 Levels

**Figure 5:**
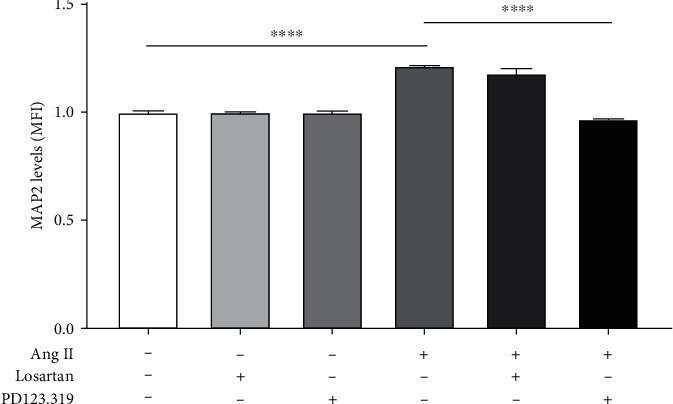
Effect of selective AT1 or AT2 receptor blockers on neuroblastoma MAP2 expression levels induced by Ang II. Cells were incubated with Ang II (600 nM) alone, Ang II plus Losartan (10^−7^ M), or Ang II plus PD123.319 (10^−7^ M) for 24 h, and the levels of MAP2 were quantified by flow cytometry. A significant increase was observed in MAP2 levels when the cells were incubated with Ang II alone compared to untreated. The levels of MAP2 decreased significantly when the cells were exposed to Ang II plus PD123.319 compared to those exposed to Ang II alone. However, no significant differences were observed when the cells were incubated with Ang II plus Losartan compared to those exposed to Ang II alone. Each bar represents the average of four experiments. Vertical lines at each bar represent SEM (^∗∗∗∗^*p* < 0.0001). MFI: mean fluorescence intensity.

We investigated first if the effects of Ang II on MAP2 were mediated through its binding to the AT1 or AT2 receptors. For this, cells were incubated with Ang II (600 nM) in the presence or absence of Losartan (10^−7^ M) or PD123.319 (10^−7^ M) for 24 h, and MAP2 levels were measured by flow cytometry. Our results showed that MAP2 levels decreased significantly when the cells were incubated with Ang II plus PD123.319 (0.97 ± 0.01 MFI; *p* < 0.0001) compared to cells incubated with Ang II alone. However, no significant changes were observed in MAP2 levels between neuroblastoma cells incubated with Ang II alone (1.22 ± 0.01 MFI) and Ang II plus Losartan (1.18 ± 0.03 MFI) (Figure 5).

**Figure 6:**
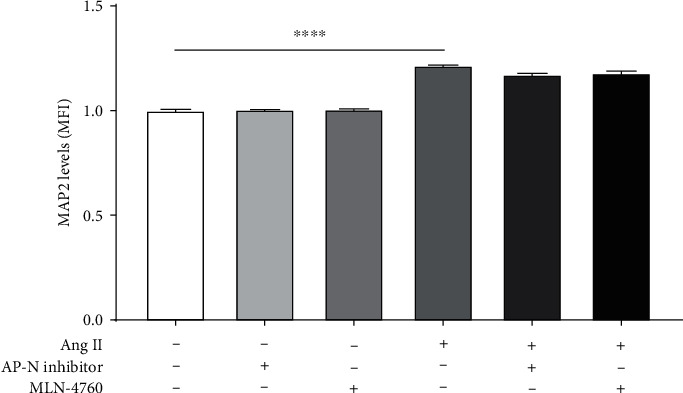
Effect of Ang II metabolism inhibitors on neuroblastoma MAP2 expression levels. Cells were incubated with Ang II (600 nM) alone, Ang II plus AP-N inhibitor (10^−7^ M), or Ang II plus MLN-4760 (10^−7^ M) for 24 h, and the levels of MAP2 were quantified by flow cytometry. A significant increase was observed on MAP2 levels when the cells were incubated with Ang II alone compared to untreated. However, no significant differences were observed in the levels of MAP2 when the cells were exposed to Ang II alone or Ang II plus AP-N inhibitor or MLN-4760. Each bar represents the average of four experiments. Vertical lines at each bar represent SEM (^∗∗∗∗^*p* < 0.0001). MFI: mean fluorescence intensity.

In other experiments, we investigated if the effects of Ang II or its metabolites on MAP2 were mediated through its binding to the AT4 or Ang-(1–7) receptors using inhibitors of Ang II metabolism. For this, cells were exposed to Ang II (600 nM) in the presence or absence of the AP-N/CD13 inhibitor (10^−7^ M) or MLN-4760 (10^−7^ M) for 24 h. No significant changes were observed in MAP2 levels when cells were exposed to Ang II alone (1.22 ± 0.01 MFI) compared to those with Ang II plus AP-N/CD13 inhibitor (1.17 ± 0.01 MFI) or Ang II plus an MLN-4760 (1.18 ± 0.02 MFI) (Figure 6).

## 4. Discussion

The many roles of Ang II in the brain remain unclear. The peptide is considered a mediator of oxidative stress activation that may contribute to neuroinflammation, but its actions in other systems can go from vasoconstriction, sympathetic activation, sodium and fluid retention, cardiac remodeling, cell growth, and cell differentiation [20–22]. Although the activation of oxidative stress induced by Ang II has been studied in different mammalian cells, there is no clear data about the mechanism employed by Ang II during neuroblastoma differentiation and their relationship with ROS generation.

In our study, we exposed human neuroblastoma cells to Ang II at 600 nM, and morphological changes in size and shape were interestingly observed under the microscope and confirmed by flow cytometry when the cells were incubated with Ang II for a period of 24 h. The cell soma size was reduced by Ang II treatment, but also, neurites extending from the cell body were observed, suggesting an activation of cell differentiation. To confirm that Ang II was able to induce neuroblastoma differentiation, we analyzed the expression of MAP2 (a marker of neuronal differentiation that stabilizes microtubules in the dendrites of postmitotic neurons) [23, 24] and NF-H subunit (a marker of neuronal differentiation required for the radial growth of axons) [25, 26]. Our data revealed a significant increase in MAP2 expression levels when neuroblastoma cells were incubated with Ang II for 24 h; however, no significant changes were observed in NF-H levels when compared to untreated cells. These results are consistent with other studies that use MAP2 levels to monitor the effects of Ang II in neuronal differentiation. As an example, Ang II was able to promote neuronal differentiation in PC12W cells, a clone of the pheochromocytoma tumor of the rat adrenal medulla used in several studies to monitor neuronal differentiation. Differentiation of rat PC12W cells to neurons was confirmed by increased neurite elongation [27] and polymerized *β*-tubulin and MAP2 levels [9].

In addition, we observed that Ang II at 600 nM increased significantly the intracellular ROS levels during differentiation of neuroblastoma cells after incubation for 24 h. It is important also to say that no cell death was observed during this period. Thus, the possibility that ROS may act as secondary messengers responsible for the differentiation of neuroblastoma cells cannot be ruled out [28, 29]. Li et al. reported that ROS produced by NADPH oxidase isoform Nox4 activate p38-MAPK and drive nuclear translocation of the cardiac transcription factor myocyte enhancer factor 2C (MEF2C) leading to cardiomyocyte differentiation in CGR8 cells [28]. Moreover, Xiao et al. demonstrated that Nox4-derived ROS mediate the differentiation of ES-D3 cells, a clonal embryonic mouse stem cells derived from blastocysts, into smooth muscle cells by promoting upregulation of the expression and phosphorylation of serum response element (SRF) and driving the translocation of SRF into nucleus for gene transcription [29].

In our study, we also investigated if the neuroblastoma ROS production induced by Ang II was mediated through NADPH oxidase. We observed that the levels of ROS induced by Ang II decreased when the cells were incubated with the NADPH oxidase inhibitor, apocynin. Numerous studies have focused on the role of the NADPH oxidase in intracellular ROS production. Studies in vascular cells have shown that ROS generation induced by Ang II involves the activation of signaling pathways that include the NADPH oxidase [30], protein kinase C (PKC) [31], and Rac-1 [32]. Lijnen et al. also demonstrated that Ang II increases superoxide anion and ROS production in cardiac fibroblasts through NADPH oxidase activation, an effect that was abrogated by apocynin as well [33]. In addition, Thakur et al. showed that acute Ang II stimulation (100 nM for 30 min) promotes ROS production by NADPH oxidase isoform Nox2 in endothelial cells isolated from mice [34]. Other studies in astrocyte cells revealed that Ang II increases ROS and superoxide levels via NADPH oxidase in a concentration- and time-dependent manner [12]. Our results are comparable with those studies due to the fact that NADPH oxidase inhibition with apocynin reduced the ROS levels that were increased by Ang II in differentiated neuroblastoma cells.

Our data also supports that activation of NADPH oxidase by Ang II was involved in the differentiation of neuroblastoma cells. This was confirmed when we analyzed the levels of MAP2 in cells exposed to Ang II plus the drug apocynin. Although we found that ROS and MAP2 levels were increased simultaneously by Ang II through the activation of NADPH oxidase, the mechanism employed by ROS during neuroblastoma differentiation needs more investigation.

In our study, we also investigated the type of angiotensin II receptor (ATR) involved in neuroblastoma differentiation. For this, we used AT1 or AT2 receptor blockers or Ang II metabolism inhibitors to antagonize the possible effects of the peptide or its metabolites to their corresponding receptors. We observed that the levels of MAP2 induced by Ang II decreased when the cells were incubated with the AT2 receptor blocker (PD123.319) with no changes when they were exposed to the peptide plus Losartan (AT1R blocker), AP-N inhibitor, or ACE2 inhibitor. Although most of the known biochemical and cellular responses to Ang II in numerous tissues have been found to be mediated by the AT1 receptor, the actions of the peptide and its metabolites in the brain have not yet been defined. Studies have shown that the AT2 receptor is widely expressed in the fetoplacental unit, but is found at very low levels in adult tissues [35]. The high abundance of this receptor is also transiently expressed in rodent fetus, suggesting that Ang II could have a role in the AT2 receptor during development and cell differentiation [36]. For example, activation of this receptor can induce neurite formation and neuronal differentiation *in vitro* from rat pheochromocytoma cell line PC12W and fetal rat neurons [9, 37]^,^revin [38]. In humans, mutations of the AT2 receptor have been found in female or male patients with mental retardation and have been implicated in brain development and maturation [39, 40].

## 5. Conclusion

Our findings support the importance of understanding the mechanisms employed by Ang II on neuroblastoma differentiation and ROS generation that may contribute to develop new therapeutic approaches to promote neuroregeneration in pathological conditions.

## Data Availability

Data supporting the findings of this study are available from the corresponding author upon request.

## References

[1] Jackson L., Eldahshan W., Fagan S. C., Ergul A. (2018). Within the brain: the renin angiotensin system. *International Journal of Molecular Sciences*.

[2] Bodiga V. L., Bodiga S. (2013). Renin angiotensin system in cognitive function and dementia. *Asian Journal of Neuroscience*.

[3] Mogi M., Iwanami J., Horiuchi M. (2012). Roles of brain angiotensin II in cognitive function and dementia. *International Journal of Hypertension*.

[4] Ganguly G., Chakrabarti S., Chatterjee U., Saso L. (2017). Proteinopathy, oxidative stress and mitochondrial dysfunction: cross talk in Alzheimer’s disease and Parkinson’s disease. *Drug Design, Development and Therapy*.

[5] Wu L., Jia Z., Yan L. (2013). Angiotensin II promotes cardiac differentiation of embryonic stem cells via angiotensin type 1 receptor. *Differentiation*.

[6] Zheng X., Wu Y., Zhu L. (2013). Angiotensin II promotes differentiation of mouse embryonic stem cells to smooth muscle cells through PI3-kinase signaling pathway and NF-*κ*B. *Differentiation*.

[7] Kim S., Zingler M., Harrison J. K. (2016). Angiotensin II regulation of proliferation, differentiation, and engraftment of hematopoietic stem cells. *Hypertension*.

[8] Gallinat S., Csikos T., Meffert S., Herdegen T., Stoll M., Unger T. (1997). The angiotensin AT2 receptor down-regulates neurofilament M in PC12W cells. *Neuroscience Letters*.

[9] Stroth U., Meffert S., Gallinat S., Unger T. (1998). Angiotensin II and NGF differentially influence microtubule proteins in PC12W cells: role of the AT_2_ receptor. *Brain Research Molecular Brain Research*.

[10] el Bekay R., Álvarez M.´., Monteseirín J. (2003). Oxidative stress is a critical mediator of the angiotensin II signal in human neutrophils: involvement of mitogen-activated protein kinase, calcineurin, and the transcription factor NF-*κ*B. *Blood*.

[11] Jaimes E. A., Galceran J. M., Raij L. (1998). Angiotensin II induces superoxide anion production by mesangial cells. *Kidney International*.

[12] Liu G., Hosomi N., Hitomi H. (2011). Angiotensin II induces human astrocyte senescence through reactive oxygen species production. *Hypertension Research*.

[13] Tsatmali M., Walcott E., Makarenkova H., Crossin K. L. (2006). Reactive oxygen species modulate the differentiation of neurons in clonal cortical cultures. *Molecular and Cellular Neurosciences*.

[14] Tsatmali M., Walcott E. C., Crossin K. L. (2005). Newborn neurons acquire high levels of reactive oxygen species and increased mitochondrial proteins upon differentiation from progenitors. *Brain Research*.

[15] Katoh S., Mitsui Y., Kitani K., Suzuki T. (1997). Hyperoxia induces the differentiated neuronal phenotype of PC12 cells by producing reactive oxygen species. *Biochemical and Biophysical Research Communications*.

[16] Sukhanov S., Yoshida T., Michael Tabony A. (2011). Angiotensin II, oxidative stress and skeletal muscle wasting. *The American Journal of the Medical Sciences*.

[17] Wen H., Gwathmey J. K., Xie L. (2012). Oxidative stress-mediated effects of angiotensin II in the cardiovascular system. *World Journal of Hypertension*.

[18] Kazama K., Anrather J., Zhou P. (2004). Angiotensin II impairs neurovascular coupling in neocortex through NADPH oxidase-derived radicals. *Circulation Research*.

[19] Sun C., Sellers K. W., Sumners C., Raizada M. K. (2005). NAD(P)H oxidase inhibition attenuates neuronal chronotropic actions of angiotensin II. *Circulation Research*.

[20] Benigni A., Cassis P., Remuzzi G. (2010). Angiotensin II revisited: new roles in inflammation, immunology and aging. *EMBO Molecular Medicine*.

[21] Zhu Y. C., Zhu Y. Z., Lu N., Wang M. J., Wang Y. X., Yao T. (2003). Role of angiotensin AT1 and AT2 receptors in cardiac hypertrophy and cardiac remodelling. *Clinical and Experimental Pharmacology & Physiology*.

[22] Chung O., Kühl H., Stoll M., Unger T. (1998). Physiological and pharmacological implications of AT1 versus AT2 receptors. *Kidney International Supplement*.

[23] Zhao F., Wu T., Lau A. (2009). Nrf2 promotes neuronal cell differentiation. *Free Radical Biology & Medicine*.

[24] Soltani M. H., Pichardo R., Song Z. (2005). Microtubule-associated protein 2, a marker of neuronal differentiation, induces mitotic defects, inhibits growth of melanoma cells, and predicts metastatic potential of cutaneous melanoma. *The American Journal of Pathology*.

[25] Hanes A. (2010). *Effects of mitochondrial dysfunction on neurofilament turnover and distribution in human neuroblastoma cells*.

[26] Yuan A., Rao M. V., Veeranna, Nixon R. A. (2012). Neurofilaments at a glance. *Journal of Cell Science*.

[27] Meffert S., Stoll M., Steckelings U. M., Bottari S. P., Unger T. (1996). The angiotensin II AT_2_ receptor inhibits proliferation and promotes differentiation in PC12W cells. *Molecular and Cellular Endocrinology*.

[28] Li J., Stouffs M., Serrander L. (2006). The NADPH oxidase NOX4 drives cardiac differentiation: role in regulating cardiac transcription factors and MAP kinase activation. *Molecular Biology of the Cell*.

[29] Xiao Q., Luo Z., Pepe A. E., Margariti A., Zeng L., Xu Q. (2009). Embryonic stem cell differentiation into smooth muscle cells is mediated by Nox4-produced H2O2. *American Journal of Physiology Cell Physiology*.

[30] Nguyen Dinh Cat A., Montezano A. C., Burger D., Touyz R. M. (2013). Angiotensin II, NADPH oxidase, and redox signaling in the vasculature. *Antioxidants & Redox Signaling*.

[31] Wang G., Anrather J., Glass M. J. (2006). Nox2, Ca^2+^, and protein kinase C play a role in angiotensin II-induced free radical production in nucleus tractus solitarius. *Hypertension*.

[32] Zimmerman M. C., Dunlay R. P., Lazartigues E. (2004). Requirement for Rac1-dependent NADPH oxidase in the cardiovascular and dipsogenic actions of angiotensin II in the brain. *Circulation Research*.

[33] Lijnen P., Papparella I., Petrov V., Semplicini A., Fagard R. (2006). Angiotensin II-stimulated collagen production in cardiac fibroblasts is mediated by reactive oxygen species. *Journal of Hypertension*.

[34] Thakur S., du J., Hourani S., Ledent C., Li J. M. (2010). Inactivation of Adenosine A_2A_ Receptor Attenuates Basal and Angiotensin II-induced ROS Production by Nox2 in Endothelial Cells. *Journal of Biological Chemistry*.

[35] Schütz S., Le Moullec J. M., Corvol P., Gasc J. M. (1996). Early expression of all the components of the renin-angiotensin-system in human development. *The American Journal of Pathology*.

[36] Millan M. A., Jacobowitz D. M., Aguilera G., Catt K. J. (1991). Differential distribution of AT1 and AT2 angiotensin II receptor subtypes in the rat brain during development. *Proceedings of the National Academy of Sciences of the United States of America*.

[37] Li J. M., Mogi M., Tsukuda K. (2007). Angiotensin II-induced neural differentiation via angiotensin II type 2 (AT2) receptor-MMS2 cascade involving interaction between AT2 receptor-interacting protein and Src homology 2 domain-containing protein-tyrosine phosphatase 1. *Molecular Endocrinology*.

[38] Danigo A., Rovini A., Bessaguet F. (2021). The angiotensin II type 2 receptor, a target for protection and regeneration of the peripheral nervous system?. *Pharmaceuticals*.

[39] Vervoort V. S., Beachem M. A., Edwards P. S. (2002). AGTR2 mutations in X-linked mental retardation. *Science*.

[40] Ichiki T., Labosky P. A., Shiota C. (1995). Effects on blood pressure and exploratory behaviour of mice lacking angiotensin II type-2 receptor. *Nature*.

